# Peptides of Presenilin-1 Bind the Amyloid Precursor Protein Ectodomain and Offer a Novel and Specific Therapeutic Approach to Reduce ß-Amyloid in Alzheimer’s Disease

**DOI:** 10.1371/journal.pone.0122451

**Published:** 2015-04-29

**Authors:** Nazneen N. Dewji, S. Jonathan Singer, Eliezer Masliah, Edward Rockenstein, Mihyun Kim, Martha Harber, Taylor Horwood

**Affiliations:** 1 Department of Medicine, University of California San Diego, La Jolla, CA, 92093, United States of America; 2 Department of Neurosciences, University of California San Diego, La Jolla, CA, 92093, United States of America; 3 Department of Pathology, University of California San Diego, La Jolla, CA, 92093, United States of America; 4 Department of Biology, University of California San Diego, La Jolla, CA, 92093, United States of America; 5 Cenna Biosciences Incorporated, 505 Coast Boulevard, Suite 302, La Jolla, CA, 92037, United States of America; 6 FortéBio, Pall Corporation, 1360 Willow Rd, Suite 201, Menlo Park, CA, 94025, United States of America; 7 Department of Neuroscience Imaging Core, University of California San Diego, La Jolla, CA, 92093, United States of America; Massachusetts General Hospital, UNITED STATES

## Abstract

β-Amyloid (Aβ) accumulation in the brain is widely accepted to be critical to the development of Alzheimer’s disease (AD). Current efforts at reducing toxic Aβ40 or 42 have largely focused on modulating γ-secretase activity to produce shorter, less toxic Aβ, while attempting to spare other secretase functions. In this paper we provide data that offer the potential for a new approach for the treatment of AD. The method is based on our previous findings that the production of Aβ from the interaction between the β-amyloid precursor protein (APP) and Presenilin (PS), as part of the γ-secretase complex, in cell culture is largely inhibited if the entire water-soluble NH_2_-terminal domain of PS is first added to the culture. Here we demonstrate that two small, non-overlapping water-soluble peptides from the PS-1 NH_2_-terminal domain can substantially and specifically inhibit the production of total Aβ as well as Aβ40 and 42 *in vitro* and *in vivo* in the brains of APP transgenic mice. These results suggest that the inhibitory activity of the entire amino terminal domain of PS-1 on Aβ production is largely focused in a few smaller sequences within that domain. Using biolayer interferometry and confocal microscopy we provide evidence that peptides effective in reducing Aβ give a strong, specific and biologically relevant binding with the purified ectodomain of APP 695. Finally, we demonstrate that the reduction of Aβ by the peptides does not affect the catalytic activities of β- or γ-secretase, or the level of APP. P4 and P8 are the first reported protein site-specific small peptides to reduce Aβ production in model systems of AD. These peptides and their derivatives offer new potential drug candidates for the treatment of AD.

## Introduction

The scourge of Alzheimer’s disease (AD) as yet has no successful disease-modifying therapies. The few FDA-approved drugs only provide mild symptomatic relief for a short period of time [1]. The molecular pathology of the disease includes the production and accumulation of Aβ [2] in the brain. Aβ is widely regarded to be the major neurotoxic agent in the initiation of AD [3]. It is a set of 39–43 amino acid oligopeptides each of which is proteolytically cleaved from its precursor protein, APP [4], by two successive protease activities: β-secretase at the Aβ NH_2_-terminus and the γ-secretase complex at its COOH-terminus, of which Presenilin (PS)-1 or -2 is the catalytic component [5, 6].

Most of the current strategies to achieve a therapy for AD aim to reduce the effects of Aβ by modifying the activities of β- or γ-secretase to produce the less toxic shorter Aβ species instead of Aβ40 and 42. Earlier such attempts at lowering total Aβ production were unsatisfactory as they directly targeted the catalytic activities of β- or γ-secretase, enzymes known to hydrolyze other substrates as well as APP. γ-Secretase, in particular, acts upon many Type I membrane proteins [7], including Notch, to yield the Notch intracellular domain (NICD) that has critically important cellular functions [8]. Although some recent γ-secretase modulation studies have successfully spared Notch function [9,10], γ-secretase has over 50 known substrates [11], any of whose functions could potentially be undesirably affected by enzyme modulation. New therapeutic approaches that can inhibit total Aβ production without targeting the activities of the β- or the γ-secretase are therefore of great interest.

Here we provide data that offer the potential for a new, early and effective approach for the treatment of AD, based on a strategy that does not target the secretases. Our work was prompted by our previous proposal [12] and findings with cell cultures [13] that the specific binding of APP with PS is a required initial step in the production of Aβ from APP, because when the cells were cultured in media containing an excess of the water-soluble NH_2_-terminal domain of PS-1 (residues 1–80) coupled to the FLAG protein, Aβ production was markedly inhibited. In that work we had selected the NH_2_-terminal domain of PS to inhibit the PS-APP interaction based on our previous findings [14–16] of an exoplasmic orientation of the NH_2_-terminus of PS in the plasma membrane. Much controversy has surrounded the assignment of the NH_2_-terminus of PS, with evidence for both its luminal [14–17] and cytoplasmic orientations [18–23]. Recently X-ray crystallographic evidence has confirmed a 9-transmembrane domain (TMD) structure [24] of the PS homolog PSH, but this technique cannot confirm orientation. There is evidence with other polytopic proteins of more than one orientation of the NH_2_- or COOH- terminus [25,26] but whether PS similarly adopts two orientations, or whether it may adopt different orientations in the plasma membrane and endoplasmic reticulum (ER) (where most of the work showing a cytoplasmic orientation of the PS NH_2_-terminal domain has been carried out) is not known. Since we could experimentally reduce the level of Aβ in the presence of the NH_2_-terminal domain of PS-1, we decided to exploit the potential therapeutic significance of these results: to inquire whether isolated small soluble peptides from within the NH_2_-terminal domain of PS-1 exhibited enough of the inhibitory activity of the whole domain, such that when added to model systems of AD, they effectively reduced Aβ production. Our first results with this approach are herein described.

## Materials and Methods

### Peptides

Peptides P1 through P10 (Table 1) were synthesized and evaluated for purity (>98% by HPLC) by Dr. Michael Freydkin of Biopeptides, San Diego.

**Table 1:** Sequences of PS-1 NH_2_-Terminal Domain Used In Peptide Synthesis.

Name	Residues	Sequence
P1	1–80	MTELPAPLSYFQNAQMSEDNHLSNTVRSQNDNRERQEHNDRRSLGHPEPLSNGRPQGNSRQVVEQDEEEDEELTLKYGAK
SP1	S1-80	PTERLLKGTVRLKQQLDQDESDARNLRNNLNPQRGFMALDESERHENRSAMQVESHQNPHPPYETEQENDEGSGSEYSNV
P2	1–40	MTELPAPLSYFQNAQMSEDNHLSNTVRSQNDNRERQEHND
P3	41–80	RRSLGHPEPLSNGRPQGNSRQVVEQDEEEDEELTLKYGAK
P4	41–55	RRSLGHPEPLSNGRP
P5	51–65	SNGRPQGNSRQVVEQ
P6	60–74	RQVVEQDEEEDEELT
P7	66–80	DEEEDEELTLKYGAK
P8	66–73	DEEEDEEL
P9	41–46	RRSLGH
P10	47–55	PEPLSNGRP

### Analysis of endogenous Aβ production *in vitro* following peptide treatment

A human neuroblastoma cell-line, IMR-32 (ATCC CCL-127) was cultured in MEM Earles medium containing 10% fetal bovine serum, 2 mM L-glutamine in the presence of penicillin and streptomycin. Equal numbers of cells (0.3 x 10^6^ per well) were plated on 6-well plates overnight. Peptides (0–5 μM) were added to fresh culture medium (500 μl per well) and left in the incubator for 24 hours, after which the culture medium from each well was removed and stored at -80°C in the presence of 4-(2-Aminoethyl) benzenesulfonyl fluoride hydrochloride (AEBSF) and protease inhibitors. Prior to use, the culture medium was centrifuged in an eppendorf microfuge for 5 min at 4°C, the supernatant diluted with an equal volume of sample diluent buffer (Wako) and analyzed for the presence of Aβ40 or 42 using a commercial ELISA kit (Wako).

### Animals for *in vivo* studies

All studies were performed using mThy1-hAPP transgenic (Tg) mice [27], which exhibit many of the manifestations of human AD. These mice express mutated (London V717I and Swedish K670M/N671L) hAPP751 under the control of the neuronal murine (m)Thy-1 promoter. Therefore, the APP involved in the Tg mouse experiments had an ectodomain that was identical, with the exception of two amino acids at the start of the Aβ region, to that of normal human APP, whereas the PS-1 involved was normal mouse PS-1, which is 92% identical to its human counterpart. The Tg lines were maintained by crossing heterozygous Tg mice with non-Tg wild type C57BL/6 x DBA/2 F1 breeders. All animal studies were carried out according to protocols approved by the Institutional Animal Care and Use Committee of the University of California San Diego.

### Animal surgeries

Age-matched male and female mThy1-hAPP Tg mice [27] at 6 months of age were anesthetized and under sterile conditions, a 26 gauge stainless steel cannula (Alzet Brain Infusion kit 3, Durect Corp) was stereotaxically implanted into the right ventricle using the following coordinates: –1 mm using Bregma as a reference [28], lateral, 1.0 mm, depth, -3.0 mm, and secured to the cranium using superglue. The cannula was connected via a 5 mm coil of V3 Biolab vinyl tubing to a model 1002 osmotic minipump (Alzet, Durect Corp) surgically placed subcutaneously beneath the shoulder blades and surgically closed [29]. The solutions (each peptide at 10 μM in PBS) were delivered directly into lateral ventricles at a flow rate of 0.25 μl per hour (100 μl total volume) for two weeks. At the end of the two-week treatment period, the mice were maintained while the cannulae remained in the brain for an additional two weeks prior to sacrifice.

### Fixation and tissue preparation

All mice were sacrificed by deep anesthesia and cardiac perfusion with 100 ml of cold saline following NIH recommendations for humane treatment of animals. The brains were removed and the left hemibrain was immersion-fixed with 4% paraformaldehyde in PBS (pH 7.4) at 4°C for 48h. Consecutive 40 μm sagittal sections (~50–60 per animal) were cut using a Leica Vibratome 2000 (Leica, Germany) and stored in cryoprotectant solution at –20°C for subsequent immunostaining and immunohistochemical computer-aided image analysis. The right hemibrain was kept frozen at –80°C for subsequent biochemical and molecular analysis of Aβ levels and APP processing.

### Analysis of Aβ deposits

For analysis of Aβ deposits, blind-coded 40 μm vibratome sections were immunolabeled as previously described [29] with mouse monoclonal antibodies (MAb) against human Aβ (clone 82E1, prepared against Aβ 1–16) followed by incubation with fluorescein isothiocyanate (FITC)-conjugated anti-mouse IgG (Vector Laboratories). Sections were imaged with a laser scanning confocal microscope (MRC1024, BioRad) as described [30] and digital images were analyzed with the NIH Image 1.43 program to determine the percent area occupied by Aβ deposits. Three immunolabeled sections were analyzed per mouse and the average of individual measurements was used to calculate group means. All sections were processed simultaneously under the same conditions and experiments were performed twice to assess reproducibility. To confirm the specificity of primary antibodies, control experiments were performed on sections in the absence of primary antibody or with pre-immune serum and primary antibody alone.

### Quantification of Aβ levels in brain extracts by ELISA

Samples from mouse cortex and hippocampus were homogenized in ice-cold, 6 M guanidine-HCl according to published protocols [29]. Quantification of Aβ 1–40 and 1–42 was carried out by ELISA kits (Invitrogen).

### Baculovirus expression, purification and characterization of soluble APP (APPs)

#### Growth and infection of sf9 cells

Sf9 cells (ATCC CRL 1711) were grown in TMN-FH complete medium (Pharmingen) in suspension and infected at a multiplicity of infection of 2 with a titred stock of recombinant virus expressing human APP695. Conditioned media and/or cells were harvested 6 days after infection. Cells and debris were removed by centrifugation (at 1000 x g for 15 min).

#### Construction of recombinant baculovirus for expression of APP695

Recombinant baculoviruses were produced according to published protocols [31]. A fragment containing full-length APP 695 was subcloned into the baculovirus transfer vector pBlueBac3 (Invitrogen). The resulting recombinant vector was co-transfected with baculovirus DNA into sf9 cells. Recombinant APP expression was verified by Western blot hybridization using mouse MAb against human APP (Cat # MAB348, Millipore).

#### Purification of soluble APP (APPs)

APPs was purified from baculovirus conditioned medium to >95% purity, as assessed by densitometry analysis of Coomassie stained SDS PAGE gels, by Arvys Proteins, Stamford, CT, using the following strategy. The conditioned medium proteins were purified by heparin-affinity chromatography, followed by two gel filtration chromatography steps over HiPrep Sephacryl S300 columns and two steps of anion exchange chromatography on HiTrapQ columns.

### Binding affinity measurements by biolayer interferometry

The binding affinities of peptides P1, scrambled P1, P4, P5, P7 and P8 for the soluble ectodomain of APP or control IgG were measured by biolayer interferometry on an Octet RED96 System (Pall FortéBio Corp., Menlo Park, CA). This system monitors interference of light reflected from two sources (an internal reflection surface and the liquid/solid interface of a fiber optic sensor) to measure the rate of binding of molecules to the biosensor surface.

Peptides were synthesized and biotinylated at the NH_2_-terminus by Dr. Michael Freydkin at Biopeptides, Inc., San Diego, CA, and purified to >95% by HPLC. Biotinylated peptides were loaded onto Streptavidin (SA) biosensors (Pall FortéBio Corp.) at empirically determined concentrations. All affinity measurements were carried out in Kinetics Buffer (KB) (1X PBS, 0.002% Tween-20, and 0.01% BSA) at 30°C. Typically, the biosensors were pre-equilibrated in KB for 120s, loaded with a biotinylated peptide at optimal concentrations and times, quenched with 10 μg/ml biocytin in KB for 180s, brought to baseline in KB for 120s and transferred to wells containing purified APPs (0–200 nM). Multiple negative controls were run for each peptide including association buffer only (no APP) or with IgG replacing APP. The specific conditions for the interaction of each peptide with APP are shown in Table 2. Binding kinetics were calculated using the FortéBio Data Analysis v7.1 software. The association (*k*_on_) and dissociation (*k*_off_) rate constants were obtained by fitting the association and dissociation data to a 1:1 model. Binding affinity, K_D_, was calculated as *k*_off_/*k*_on_.

**Table 2:** Assay Conditions For The Interaction Of Each Peptide With APP Or IgG.

Peptide	Loaded Peptide concentration (μg/mL)	APP titration (nM)	IgG titration (nM)	Association time (s)	Dissociation time (s)
P1	1.00	3.13–200	-	120	200
SP1	1.00	3.13–200	-	120	200
P4	0.40	3.13–200	3.13–200	360	360
P5	1.00	3.13–200	3.13–200	360	360
P7	1.00	3.13–200	3.13–200	360	360
P8	0.78	1.1–12.5	1.1–12.5	120	200

### Analysis of specific binding of P4 and P8 to APP by confocal microscopy

Fibroblasts derived from mice that were rendered null for the APP gene (APP^-/-^) (Jackson Laboratories) were plated on glass-bottom microwell culture dishes (MatTek Corporation, MA, 35mm petri dish, 14mm microwell) and transfected with a pcDNA3 construct of APP695 as described elsewhere [13]. Cells were used 24 h after transfection. Similarly plated APP^-/-^ cells were used untransfected as controls.

*Peptide Treatments*: After removal of culture medium, the culture dishes were placed on ice and cells treated with each of biotinylated peptides P4 and P8 (0.5–2 μM in ice-cold culture medium) for 5 min. The cells were then washed with ice-cold PBS, fixed for 10 min with 4% p-formaldehyde and treated with fluorescently-labeled SA (Alexa Fluor, 488 conjugate, Molecular Probes) for 10 min. After washing with PBS, cells were treated with Prolong Anti-fade (Invitrogen) and sealed with a glass cover-slip for confocal microscopy.

*Confocal microscopy*: The treated cells were observed with an Olympus FV1000 spectral laser-scanning microscope using a 60x 1.42 NA objective. Cells were excited with a 488 nm laser and were detected from 500 nm to 530 nm.

### Measurement of NICD in extracts of neocortex of treated mice

Extracts of neocortex of mThy1-hAPP mice treated with P4, P8 or PBS only were electrophoresed on SDS gels and Western blotted with primary rabbit antibodies against NICD (Cat# 07–1232, Millipore) at 4°C overnight, followed by horse-radish peroxidase (HRP)-conjugated goat anti-rabbit IgG (Bio-Rad). The immunoreactive bands were detected by chemiluminescence (Thermo Scientific).

### Measurement of APP in extracts of neocortex of treated mice

After NICD detection, the nitrocellulose membranes were stripped and re-probed with a mouse MAb against APP (Cat# MAB348, Millipore) followed by HRP-conjugated goat anti-mouse IgG (Bio-Rad). The intensity of the NICD and APP protein bands was quantified using the ImageJ program (NIH).

### Measurement of BACE-1 activity in extracts of neocortex of treated mice

Extracts of neocortex of Tg mice treated with peptides P4, P8 or PBS only were prepared as described. BACE activity was determined using the SensiZyme BACE1 activity assay kit (Cat# CS1060, Sigma, Dt. Louis, MO) according to the manufacturer's instructions [32]. This kit determines BACE-1 activity that is expressed as ng/ml active BACE-1. The protease activity measurement is based on a multistep series of reactions. Briefly, BACE-1 in extracts is captured by a BACE-1 specific immobilized antibody. After washing, functionally active BACE-1 is allowed to splice a modified procaspase-3, which serves as a substrate for BACE-1 and which upon slicing becomes activated. Next, a substrate for activated modified caspase-3 is added and the subsequent color formation is monitored at 405 nm.

### Measurement of NICD in extracts of cultured Jurkat cells

Jurkat cells (Sigma) were cultured in RPMI 1640 medium supplemented with 10% FCS, 2 mM L-glutamine, penicillin and streptomycin at 37°C in the presence of 5% CO_2_. Cells were transferred to 6-well dishes (5 x 10^6^ cells per well) and treated with peptides P4, P8 and scrambled P1 (0–10 μM) or the γ-secretase inhibitor Semagacestat LY450139 (Selleck Chemicals) (0–100 nM) for 24 h. After harvesting the cells, extracts were prepared by sonication in lysis buffer (Cell Signaling Technologies) supplemented with 1mM PMSF and complete protease inhibitor cocktail (Roche). The solubilized extracts were then used in ELISA assays to determine NICD levels, as a measure of γ-secretase activity, using the PathScan Cleaved Notch 1 (Val 1744) sandwich ELISA kit (Cell Signaling Technologies) according to the manufactuer’s protocols.

### Measurement of BACE-1 activity in extracts of cultured IMR-32 cells

IMR-32 cells in culture were treated with peptides P4, P8 and scrambled peptide P1 (0–5 μM) or the BACE-1 inhibitor LY281137 (Selleck Chemicals) (0–500 nM) for 24 h. Cells were harvested and extracts prepared by sonication and solubilization in cell lysis buffer containing 1% Triton X-100 and EDTA (Cell Signaling Technologies) and supplemented with complete protease inhibitor cocktail (Roche) according to the manufacturer’s protocols. BACE-1 activity was measured as already described above using the SensiZyme BACE-1 activity assay kit.

### Statistical analysis

Analyses were carried out with the STATView 5.0 program (SAS Institute Inc., Cary, NC). Differences among means were assessed by one-way Analysis of Variance (ANOVA) with post hoc Dunnett’s [33]. For all analyses, alpha levels were set at 0.05.

## Results

### Effects of peptides P1 to P3 on Aβ production *in vitro*

**Fig 1:**
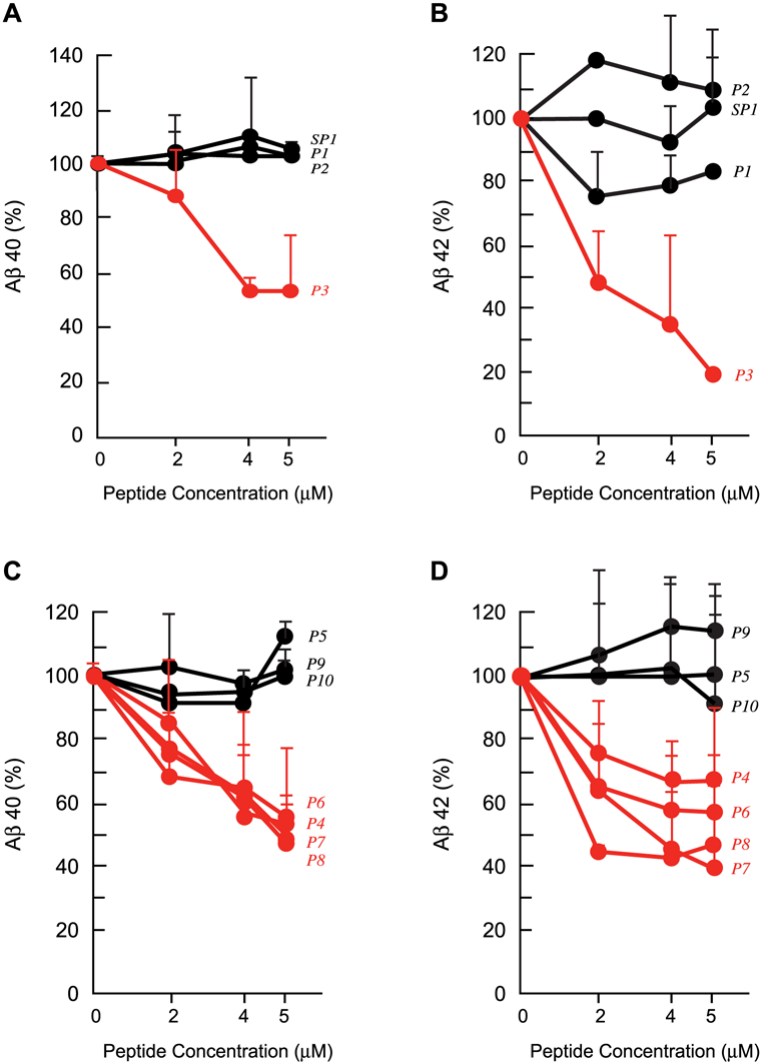
Effects Of Peptides P1 Through P10 On Aβ40 and 42 Production *In Vitro*. A and B. Peptides P1(residues 1–80 of PS-1), scrambled P1 (SP1), P2 (residues 1–40 of PS-1) and P3 (residues 41–80 of PS-1) (0–5 μM) were added to cultures of IMR-32 cells for 24 h and the culture media were analysed for Aβ40 (A) and Aβ42 (B) by ELISA. Aβ levels in pg/ml were expressed as a percentage of levels observed in the absence of added peptide. Values represent group means of 3–4 independent experiments per peptide ±s.e.m. Only P3 produced a clear dose-dependent decrease in both Aβ40 and -42, shown in red. C and D. Overlapping peptides from the P3 region (P4–P10) (0–5 μM) were next tested in IMR-32 cultures as in A and B above for their effect on Aβ40 (C) and -42 (D) production. A family of peptides, P6, P7 and P8 and an independent peptide, P4, produced substantial dose-dependent reduction in Aβ40 and 42, shown in red.

Peptide P1, comprising the first 80 amino acids of PS-1, scrambled P1 and the two large oligopeptides corresponding to the two halves of the human P1 (peptides P2 and P3, Table 1), were first synthesized and tested *in vitro* for their effect on the production of Aβ40 and 42. Peptide P3 produced a dose-dependent reduction in Aβ, with a marked and significant reduction in mean Aβ40 and 42 levels s.e.m. of 45% ± 19 (*P* = 0.05) for Aβ40 and of 80% ± 0 (*P*<0.01) for Aβ42 at 5 μM compared to no-peptide controls. Peptides P2 and scrambled P1 on the other hand produced no significant changes in Aβ levels (Fig 1A and 1B). P1 produced a small reduction in Aβ42 but the difference was not significant. This was unexpected given our previous results with the bacterially produced FLAG-PS-1 (residues1-80) [13] which produced a robust inhibition of Aβ. This difference between the synthetic and bacterially produced peptide may be due to the poorer solubility that we have observed with the synthetic peptide. Our results indicated that the peptide sequences of interest to the inhibition of Aβ production were most likely located within the P3 half of the NH_2_-terminal domain of PS-1.

### Effects of peptides P1 to P3 in Tg mouse brains

We next examined the effects of peptides P1, P2 and P3 on the production of Aβ in a transgenic (Tg) mouse model of AD [27]. These Tg mice over-express human APP751 with the London (V717I) and Swedish (K670M/N671L) mutations under the regulatory control of the neuron specific murine (m)Thy-1 promoter, line 41, (hereinafter, mThy1-hAPP). This mouse model was selected because of the high levels of Aβ42 produced in the neocortex at 3 months, and in the hippocampus at 4 months, of age [27]. mThy1-hAPP mice also exhibit relatively early appearance of synaptic damage, plaque formation and performance deficits in the water maze [27].

**Fig 2:**
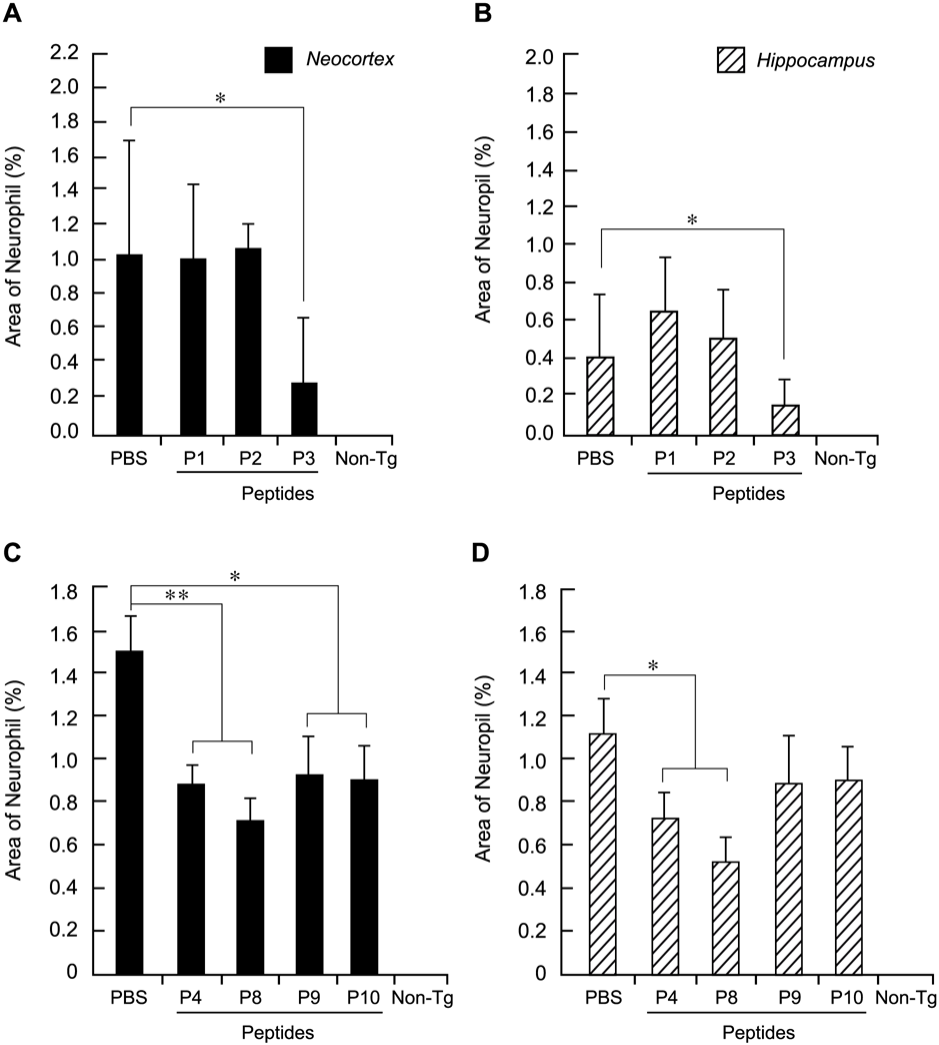
Effects Of Peptides P1 Through P10 Infusion In Brains Of mThy1-hAPP Mice on Plaque load. Effects of Peptides P1–P3 (A and B) and P4–P10 (C and D): Quantification of plaque load in neocortex (A and C) and hippocampus (B and D). Aβ- immunoreactive deposits were imaged and digital images were analyzed to determine the percent area of the neuropil occupied by Aβ. Three immunolabeled sections were analyzed per mouse and the average of individual measurements was used to calculate group means. Values represent group means ±s.e.m. *, *p*<0.05; **, *p*<0.005 versus PBS by ANOVA.

Peptides P1, P2 and P3 (10 μM solutions in PBS; this concentration was an estimated excess, based on our *in-vitro* data) were each directly delivered into the lateral ventricles of 6 month old, age-matched, male and female mThy1-hAPP mice by cannula implanted into the frontal cortex and connected to an Alzet 1002 (Durect Corp) osmotic minipump for a period of two weeks at a continuous flow rate of 0.25 μl per hour (total volume infused was 100 μl). Control mice received PBS alone. Following the termination of the two-week infusion period, the mice were maintained for a further two weeks before being sacrificed. Immunohistochemical analysis of Aβ deposits in the hippocampus and neocortex was carried out on sections of mouse brain immunolabeled with mouse MAb directed against human Aβ1–16, followed by FITC-conjugated anti-mouse IgG. Peptide P3 produced a significant reduction in mean Aβ levels ± s.e.m. of 74%±38 (*P* = 0.01, n = 3) in the neocortex (Fig 2A) and 67%±38 (*P* = 0.05, n = 3) in the hippocampus (Fig 2B) compared to PBS controls, whereas peptide P2 produced no significant changes in Aβ immunolabeling compared to controls. This confirmed the *in-vitro* data showing that the peptide sequences of interest to the inhibition of Aβ production were likely located within the P3 half of the NH_2_-terminal domain of PS-1.

### Effects of peptides P4 to P10 on Aβ production *in vitro*

We next synthesized a series of smaller overlapping peptides, P4 through P10 (Table 1), encompassing the entire P3 sequence. Our first study of these peptides employed, as before, the cell-based assay for Aβ production in IMR-32 cells. We determined the amounts of Aβ40 and Aβ42 that were produced after 24 h in the presence of varying concentrations of these synthetic peptides by Aβ40- or Aβ42- specific ELISA analysis (Fig 1C and 1D). We first examined a family of peptides that included P6, P7 and P8. P6 and P7 were overlapping peptides that each contained the DEEEDEEL sequence of P8 at their NH_2_-terminus (P7) or COOH terminus (P6) (Table 1). All three peptides produced a dose-dependent reduction in Aβ40 and 42, with the maximum reduction obtained with 5 μM peptide (Fig 1C and 1D), measured in the media of IMR-32 cells. P8 produced the maximum mean reduction ±s.e.m. of 52%±6 for Aβ40 and 51%±18 for Aβ42 with 5 μM peptide. P4, an independent peptide that was distinctly different in its composition and sequence as well as in its linear position in the PS-1 NH_2_-terminal domain from the P8 family of peptides just described, also produced a dose-dependent reduction in Aβ40 and 42 levels, with a maximum mean reduction ±s.e.m. of 48%±25 for Aβ40 and 31%±23 for Aβ42 with 5 μM peptide. Another peptide, P5, on the other hand had no significant effect on Aβ production.

### Administration of peptides P4 and P8 in brains of live Tg mice

**Fig 3:**
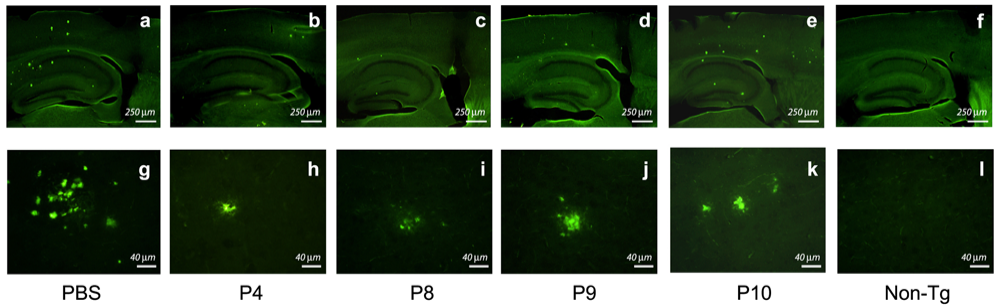
Effects Of Peptides P4 Through P10 Infusion In Brains Of mThy1-hAPP Mice. mThy1-hAPP mice were infused with either PBS or peptides for two weeks, followed by two weeks of rest, using osmotic minipumps. Fixed brains were vibratomed at 40 μm, immunolabeled with MAb against Aβ1–16, followed by FITC-conjugated anti-mouse IgG, and imaged with a scanning confocal microscope. Top Panel: Representative low-power (15x) photomicrographs of vibritome sections (bar, 250 μm). Bottom panel: High power (400x) photomicrographs of amyloid plaques in top panel (bar, 40 μm). Peptides P4 (panels b and h) and P8 (panels c and i) produced marked reductions in Aβ labeling compared to PBS controls (panels a and g).

Two independent peptides that lowered Aβ levels *in vitro*, P4 and P8, were then selected for their effects in mThy1-hAPP mouse brains. P8 was selected over P6 and P7 from that family of peptides because it was the smallest and most efficacious of the three in lowering Aβ *in vitro*. P9 and P10, two fragments of P4, which did not give a significant reduction in Aβ *in vitro*, were also examined. The same protocols that are described above for the study of peptides P1–P3 were followed, using 10 μM peptide in PBS and 6–15 mice per treatment. As before, the mice were sacrificed a total of four weeks after the start of peptide administration (two weeks of peptide treatment followed by two weeks of rest); the brains were sectioned and immunolabeled with a primary mouse MAb against human Aβ 1–16. A representative set of Aβ-labeled sections of the brains that had been treated with peptides or controls is shown (Fig 3), each at both low (top panels) and high (bottom panels) magnifications. Note first (Fig 3, panel g) the intense Aβ positive labeling of plaques in mThy1-hAPP mouse brains that received only PBS; these control mice exhibit substantial amounts of Aβ deposits in their brains by 6 months. In contrast non-Tg mice show no Aβ labeling (Fig 3, panels f and l). The different extents of Aβ labeling for the different peptides (Fig 3, panels h-k, and at lower magnification Fig 3, panels b-e) in these figures is striking. Peptides P4 and P8 produced marked reductions in Aβ labeling compared to PBS controls, whereas P9 and P10 were less effective.

These qualitative Aβ immunofluorescence results were also quantified as previously described [27,29], to determine the percent area of the neuropil that was occupied by Aβ (Aβ load). The data for the neocortex (Fig 2C) and for the hippocampus (Fig 2D) were closely similar to one another. P4, and more so P8, produced marked, statistically significant reductions in Aβ load (mean reduction in Aβ load ± s.e.m. 41%±6 for P4, *P* = 0.004; 52%±7 for P8, *P*<0.001 in the neocortex and 35%±10 for P4, *P* = 0.04; 53%±10 for P8, *P* = 0.006 in the hippocampus, n = 9 for all treatments) compared to PBS controls (Fig 2C and 2D). P9 and P10 were less effective but still produced statistically significant reductions in Aβ levels compared to PBS controls in the neocortex. P9 and P10, which are fragments of P4, had not shown a reduction of Aβ *in vitro* (Fig 1C and 1D), contrary to expectation, and we had concluded that P4, when fragmented into P9 and P10 lost its inhibitory activity. They were therefore included in these studies as negative controls. However, we did see a modest decrease in Aβ levels (compared to P4 and P8) in the *in vivo* studies, possibly due to the much longer exposure and higher concentration of the peptides.

**Fig 4:**
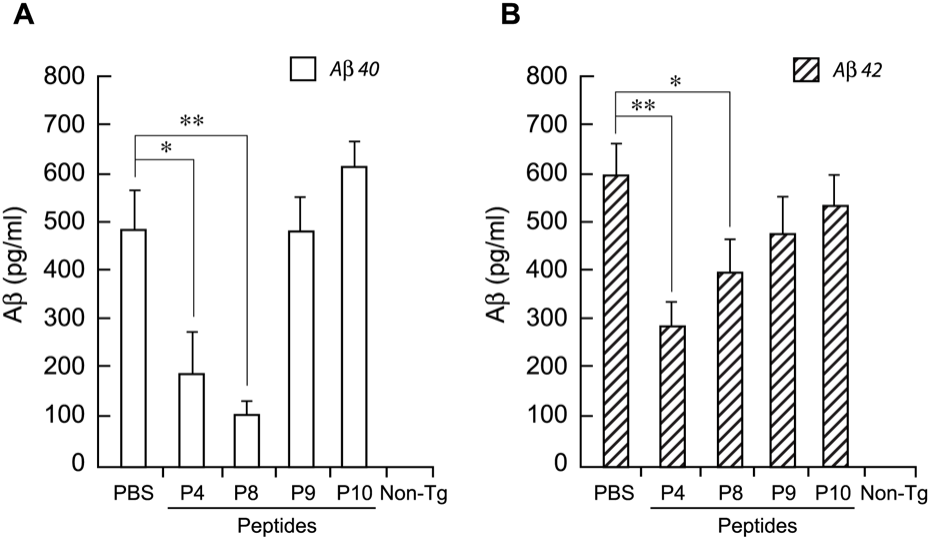
ELISA Analysis Of Aβ40 And 42 In mThy1-hAPP Mouse Brains Treated With Peptides P4 To P10. Extracts of neocortex from peptide-treated Tg mice were subjected to Aβ40- (A) and Aβ42-specific (B) ELISA analysis. Three to six samples were analyzed per mouse and the average of individual measurements was used to calculate group means. Values represent group means ±s.e.m. *, *p*<0.05; **, *p*<0.005 versus PBS, by ANOVA.

Further analysis of Aβ production in the mThy1-hAPP mouse brains was carried out by Aβ40- and 42-specific ELISA experiments. Extracts of neocortex from the peptide-treated mice were subjected to ELISA analysis (Fig 4). (The experiments in Fig 2C and 2D were performed using a mouse MAb against human Aβ 1–16, which measures total Aβ but does not discriminate between Aβ40 and 42. The experiments described in Fig 4 accomplished this discrimination). P4 and P8 showed significant reductions in the production of both Aβ40 (Fig 4A) (mean Aβ, pg/ml ± s.e.m: a reduction of 60%±17 with P4, *P* = 0.03; 77%±7 with P8, *P* = 0.002; n = 8 for all treatments) and Aβ42 (Fig 4B) (a reduction of 52%±8 with P4, *P* = 0.001 and 33%±11 with P8, *P* = 0.049; n = 9 for all treatments) compared with PBS controls; P9 and P10 also gave reductions in Aβ load, but to a lesser extent.

### On the mechanism of Aβ reduction by the peptides

The work described in this paper was based on our previous studies which had demonstrated that a peptide consisting of the first 80 amino acids of PS-1 could inhibit the production of Aβ, possibly by competing with PS-1 for binding to APP. In order to consider whether the peptides in the current study may reduce Aβ by competitively inhibiting a PS-1:APP interaction, it was important to first demonstrate that the peptides that reduced Aβ could specifically bind APP. We examined the specific interaction of select peptides with the APP ectodomain by two methods, by biolayer interferometry and by confocal microscopy.

#### Kinetics of P1–P8 binding to the APP ectodomain

**Fig 5:**
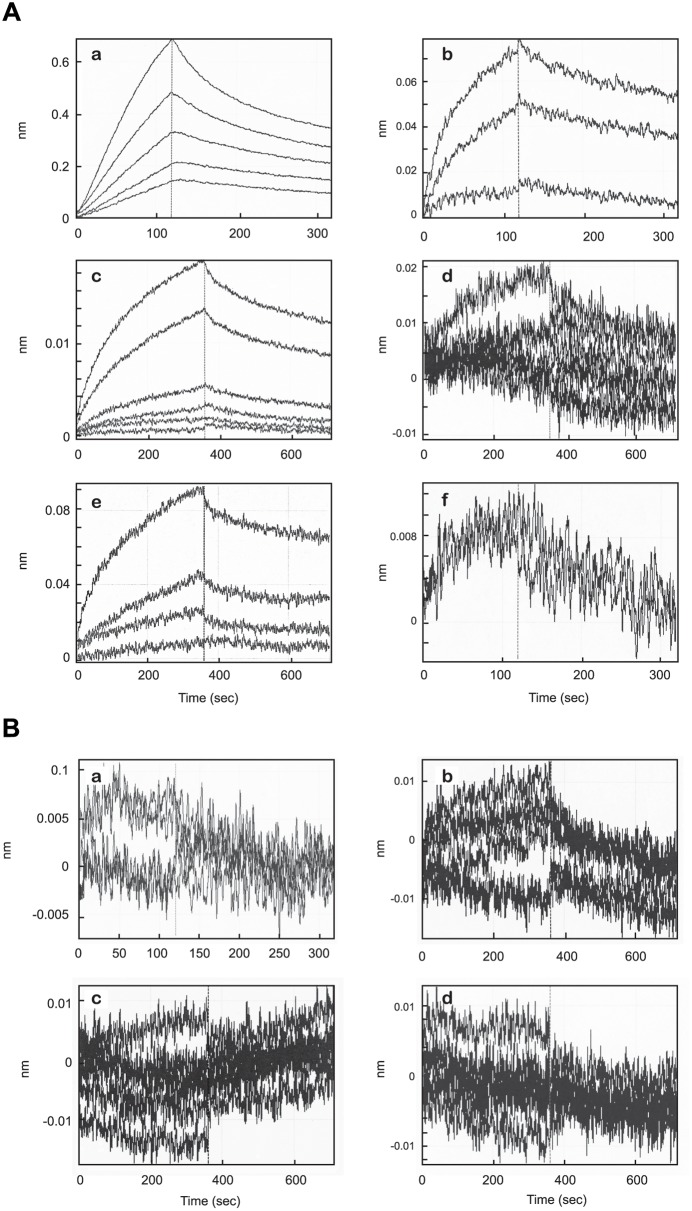
Global Kinetic Analysis Of Purified Soluble APP (A) Or Control IgG (B) Binding To Biotinylated Peptides By Biolayer Interferometry. A. Biolayer interferometry analysis of peptides with APPs. Biotinylated peptides were immobilized on SA biosensors and affinity constants (K_D_, *k*_on_, and *k*_off_) for APPs were determined in KB at 30°C. Binding curves showing association and dissociation to various concentrations of APPs of: P8 (a), P1 (b), P4 (c), P5 (d), P7 (e) and scrambled P1 (f). The times and concentrations for each sample were optimized experimentally. Each data set was fit globally with a 1:1 binding model. B. Biolayer interferometry analysis of peptides with control IgG. Biotinylated peptides were immobilized on SA biosensors. The affinity constants (K_D_, *k*_on_, and *k*_off_) for control IgG were determined in KB at 30°C with P8 (a), P7 (b), P4 (c), P5 (d). The times and concentrations for each sample was optimized experimentally. Negative data sets did not fit any binding model with confidence.

The binding affinities of serially diluted APPs to biotinylated synthetic peptides P1, scrambled P1, P4, P5, P7 and P8 immobilized on SA biosensors were measured by biolayer interferometry. The association and dissociation curves for the binding affinity measurements of peptides to APPs are shown in Fig 5A. To ensure specificity of binding, binding assays were carried out for each peptide with similar concentrations of mouse IgG (Fig 5B). Biotinylated peptides P8, P4, P7 and P1 (Fig 5A, panels a, c, e and b respectively) bound to purified APPs in a concentration-dependent manner. The binding profile of biotinylated-P8 to APPs demonstrated strong, biologically relevant, and specific affinity (K_D_) of 3.45 nM, *k*_on_ of 8.86 x 10^5^ M^-1^s^-1^, and *k*_off_ of 3.06 x 10^-3^ s^-1^ (Fig 5A, a, Table 3), with a coefficient of determination, R^2^, of 0.985, giving a high goodness of fit to a 1:1 binding model. Comparable data were obtained for the binding of biotinylated P4 and P7, where the P4:APPs interaction gave a K_D_ of 16.1 nM, *k*_on_ of 8.36 x 10^4^ M^-1^s^-1^, and *k*_off_ of 1.34 x 10^-3^ s^-1^ (Fig 5A, c, Table 3), and the P7:APPs interaction gave a K_D_ of 10.1 nM, *k*_on_ of 1.04 x 10^5^ M^-1^s^-1^, and *k*_off_ of 1.05 x 10^-3^ s^-1^ (Fig 5A, e, Table 3). Binding was also measured for P1, which includes all the sequences present in the other peptides, to APPs. The P1:APPs interaction resulted in a K_D_ of 14.3 nM, *k*_on_ of 1.20 x 10^5^ M^-1^s^-1^, and *k*_off_ of 1.72 x 10^-3^ s^-1^ (Fig 5A, b, Table 3). The binding data for P1, as reported earlier, were not as good as for the other peptides, probably, as previously suggested, due to the poorer solubility encountered for this synthetic peptide (see also Fig 1A and 1B). As shown by the results above and in Table 3, P8 showed the strongest affinity for APPs, almost 5 times stronger than that demonstrated by P4. P8 and P7 (which contains the P8 domain) on the other hand, showed a small but insignificant difference in binding affinities [34]. P5 and scrambled P1, used in these experiments as negative controls, showed no significant binding to APP under the same conditions (Fig 5A, d, f and Fig 5B, d). Specificity of the peptide binding to APPs was demonstrated by a lack of significant binding to identical concentrations of mouse IgG, used in these experiments as a control irrelevant protein. (Fig 5B, panels a-d)

**Table 3:** Kinetic Rate Constants And Affinities Of Peptide Binding To Purified APP Ectodomain Using Streptavidin Biosensors.

Peptide	K_D_ (M)	*k*_on_ (M^-1^s^-1^)	*k*_off_ (s^-1^)	Full X^2^	Full R^2^
P1	1.43E-08	1.20E+05	1.72E-03	0.004	0.991
P4	1.61E-08	8.36E+04	1.34E-03	0.065	0.995
P7	1.01E-08	1.04E+05	1.05E-03	0.041	0.978
P8	3.45E-09	8.86E+05	3.06E-03	0.537	0.985

### Specific binding of P4 and P8 to APP by confocal microscopy

**Fig 6:**
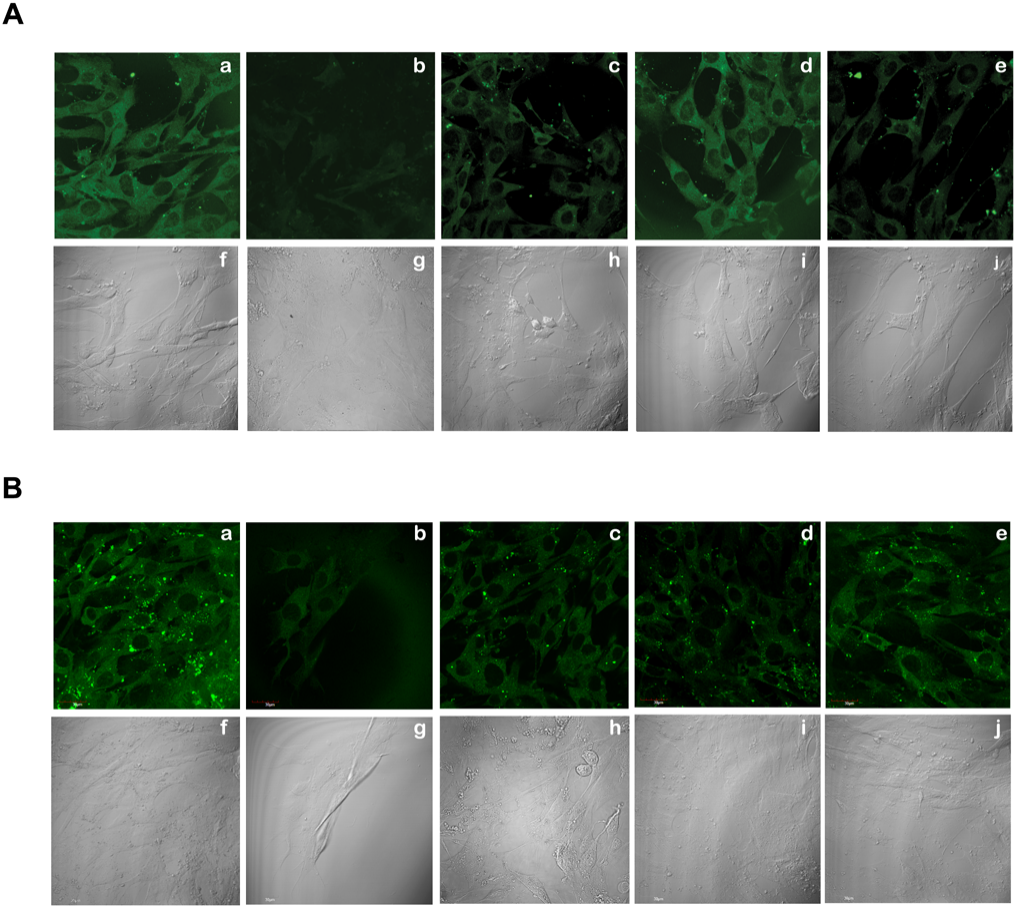
Specific Binding Of Peptides P4 And P8 To Cell-Surface APP By Confocal Microscopy. Biotinylated P4 (A) and P8 (B) were added to APP-transfected fibroblasts (panels a) or untransfected APP-null cells (panels b) and showed binding only to APP-expressing cells. Panels c in A and B show that pre-treatment of the biotinylated peptides with APP significantly reduced their binding to APP-expressing cells, compared to the untreated peptides in panels a. In A, panel d, treatment of APP-expressing cells with excess unbiotinylated P8 did not significantly alter the fluorescence observed when these cells were then treated with biotinylated P4. However, in B, panel d, when the APP-expressing cells were treated with biotinylated P8 following the same treatment, the fluorescence signal was significantly diminished compared to panel a. Similarly, when APP-expressing cells were pre-treated with excess unbiotinylated P4 prior to treatment with the biotinylated peptides, the binding of biotinylated P4 (A, panel e) but not biotinylated P8 (B, panel e) was reduced.

While clear specific binding of peptides P4, P7 and P8 to purified APPs was shown by biolayer interferometry, it was important to establish that the peptides could also bind APP expressed at the plasma membrane of live cells. We carried out our studies on APP-null mouse fibroblasts [13] that were either used after transfection with full-length human APP 695 (APP-expressing cells) or as APP-null controls. If both types of cells were treated with biotinylated peptides, followed by fluorescently-labeled SA, the peptides should only decorate the APP-expressing cells and not the controls. Results in Fig 6 show that biotinylated P4 (Fig 6A) and P8 (Fig 6B) both labeled APP-transfected fibroblasts (panels a) but not the untransfected APP-null cells (panels b), suggesting that the peptides were binding APP present on the surfaces of the APP-expressing cells.

To confirm that the peptides were indeed binding to APP and not to another protein that may be expressed because of the presence of APP, the biotinylated peptides were first treated with purified APPs (5 μM) to block the APP binding sites and the blocked peptides were then used to treat the APP-expressing cells in the usual way. Panels c in Fig 6A and 6B show that pre-treatment of the biotinylated peptides with APPs significantly reduced their binding to APP-expressing cells, compared to the untreated peptides in panels a, strongly indicating that the peptides were indeed binding APP.

We next determined whether the binding of P4 and P8 to APP occurred at the same or at distinct sites on the APP ectodomain. APP-expressing cells were treated with 2-fold excess unbiotinylated P4 or P8 for 5 min, followed by treatment with the biotinylated peptides in the usual way. In Fig 6A, panel d, treatment of APP-expressing cells with excess unbiotinylated P8 did not significantly alter the fluorescence observed when these cells were then treated with biotinylated P4. However, in Fig 6B, panel d, when the APP-expressing cells were treated with biotinylated P8 following the same treatment, the the fluorescence signal was significantly diminished compared to panel a. Similarly, when APP-expressing cells were pre-treated with excess unbiotinylated P4 prior to treatment with the biotinylated peptides, the binding of biotinylated P4 (Fig 6A, panel e) but not biotinylated P8 (Fig 6B, panel e) was reduced. These results suggest an absence of competition between P4 and P8 for binding to APP and indicate that the two peptides must bind APP at different sites on the APP ectodomain.

### On the specificity of the P4 and P8 reductions in Aβ load

**Fig 7:**
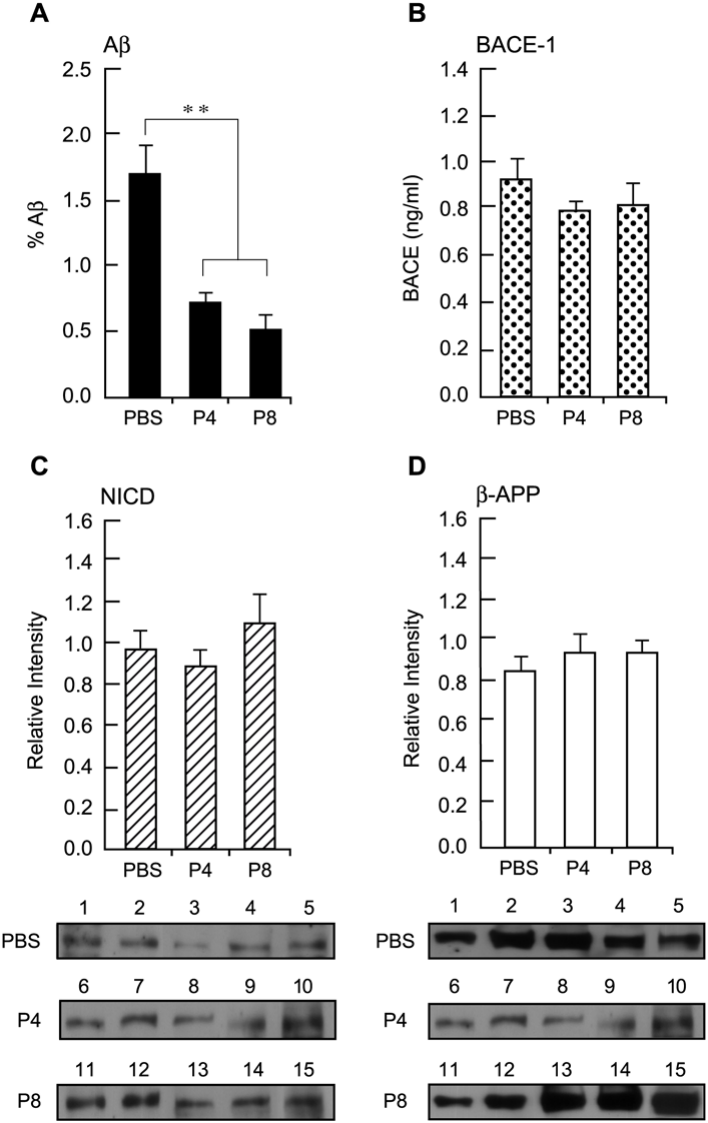
Effect Of Peptides P4 And P8 Treatment On Levels Of Aβ, APP, NICD And BACE-1 In Neocortex Of APP Tg Mice. A. Aβ levels in sections of mouse neocortex that was immunolabeled with a MAb against human Aβ1–16 were quantified as in Fig 2. Data are expressed as mean ±s.e.m. n = 5. ** *p* <0.005. B. BACE-1 activity in extracts of neocortex of mice treated with peptides P4, P8 and PBS was determined using the SensiZyme BACE1 activity assay kit. Absorbance was monitored at 405 nm. Data are expressed as mean ±s.e.m. of active BACE-1 in ng/ml n = 5. C. Extracts of neocortex of peptide-treated mice were Western-blotted with primary rabbit Ab against NICD followed by HRP-conjugated goat anti-rabbit IgG. Immunoreactive bands were detected by ECL and the signal intensity of the protein bands was quantified. Top: data are expressed as mean ±s.e.m. n = 5. Bottom: The individual Western blot gel images showing immunoreactive bands. D. After NICD detection, the nitrocellulose membranes were stripped and re-probed with a MAb against APP, followed by HRP-conjugated goat anti-mouse IgG. The signal intensity of the protein bands was then quantified. Top: data are expressed as mean ±s.e.m. n = 5. Bottom: The individual Western blot gel images showing immunoreactive bands.

An optimal therapeutic approach to AD would avoid producing serious effects on other biochemically and physiologically important processes, as would occur if the cleavages of any of the other substrates of β- or γ-secretase were affected in the process of reducing the levels of Aβ. An especially well-studied example is the γ-secretase activity of PS-1 that is critically involved in the final enzymatic cleavage of Aβ from APP; it also functions in an essential developmental process that generates NICD from Notch [35]. In order to determine whether P4 and P8 could reduce Aβ load without affecting the activities of either β- or γ-secretase, we investigated (Fig 7), as a measure of γ-secretase activity, the production of NICD in the same samples of mThy1-hAPP mouse brains that had been administered P4 and P8 for the analysis of Aβ. Samples were selected that had previously shown a reduction in Aβ load of 50% or more with the two peptides. At the same time we analyzed the same tissue samples for their β-secretase (BACE-1) activity and APP expression. Extracts were prepared of the neocortex of Tg mice that had been treated with P4, P8 or PBS, and the levels of NICD were determined by Western blot analysis with a primary rabbit Ab against NICD. This antibody recognizes only the NICD that is released after cleavage by γ-secretase. The marked reduction in Aβ levels produced by P4 (total Aβ mean reduction ± s.e.m. 58%±4, *P*<0.001) and P8 (70%±6, *P*<0.001) compared to PBS controls (n = 5, each treatment) (Fig 7A)) were not accompanied by any significant changes in the levels of NICD (Fig 7C), demonstrating that the γ-secretase activity in these samples was not measurably altered. Similarly, BACE-1 activity in the same samples, measured using an ELISA kit [32], was not significantly changed in the P4- and P8-treated samples (Fig 7B). APP levels in the same samples were also determined by Western blot analysis with a primary mouse MAb against human APP, followed by densitometric scanning of the bands, and did not show significant differences (Fig 7D).

**Fig 8:**
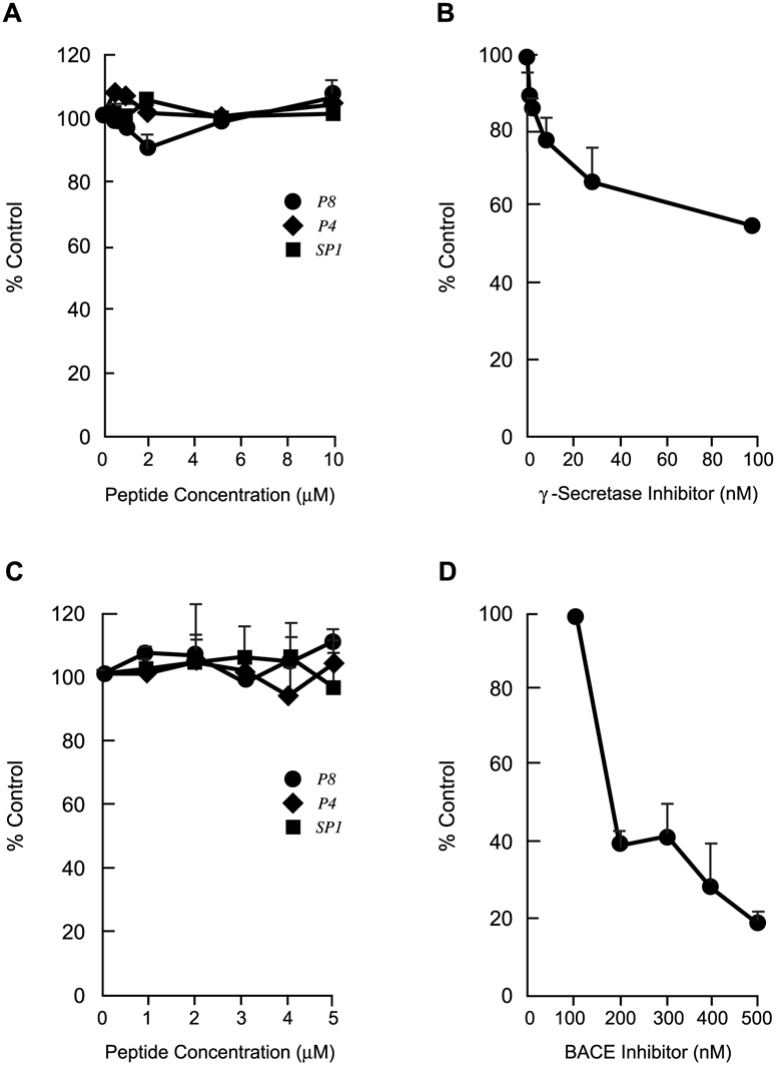
Dose-dependent Effects Of Peptides P4, P8 and Scrambled P1 On NICD Levels And on BACE-1 Activity *In Vitro*. A. NICD levels in extracts of Jurkat cells that had been treated with 0–10 μM P4, P8 and SP1 for 24 h determined by ELISA. Absorbance was monitored a 450nm and data are expressed as means ± s.e.m. of percent control without peptide. N = 2–3. B. NICD levels in extracts of Jurkat cells that had been treated with the γ-secretase inhibitor Semagacestat LY450139 for 24 h. Data are expressed as in A, n = 2. C. BACE-1 activity in extracts of IMR-32 cells that were treated with 0–5 μM P4, P8 and SP1 for 24h, determined with the SensiZyme BACE-1 activity assay kit. Absorbance was monitored at 405 nm and data are expressed as means ± s.e.m. of percent control (without peptide) BACE activity. N = 2–3. D. BACE-1 activity in extracts of IMR-32 cells that were treated with the BACE inhibitor LY281137 (0–500 nM) for 24 h, expressed as in C. N = 2.

Since the NICD levels and BACE activity in mouse brains were measured two weeks after treatment with the peptides had stopped, which theoretically could allow the inhibited β- or γ-secretase activities to return to normal, experiments were carried out *in vitro* to determine the effect of increasing concentrations of peptides P4, P8 and control scrambled P1 on the activities of the two enzymes. The results in Fig 8A show that there was no appreciable change in the levels of NICD released in extracts of Jurkat cells that were treated with increasing concentrations of P4, P8 and scrambled P1 for 24 h, whereas NICD released in these cells was inhibited in a dose-dependent manner after treatment with the γ-secretase inhibitor Semagacestat under the same conditions (Fig 8B). Jurkat cells were used in these experiments instead of IMR-32 cells as the latter express very low levels of Notch 1 [36]. Since Jurkat cells had not hereto been tested with our peptides, the highest peptide concentration used for these cells was twice that used for IMR-32 cells, to allow for any possible inhibition of γ-secretase activity at the higher peptide concentration. Similarly, BACE-1 activity measured in extracts of IMR-32 cells that were treated with increasing concentrations of peptides P4, P8 and control scrambled P1 for 24h remained essentially unchanged (Fig 8C). On the other hand the activity of the enzyme decreased in a dose-dependent fashion when treated with the β-secretase inhibitor LY281137 under the same conditions (Fig 8D). These results therefore demonstrate the absence of a measurable effect on the catalytic activities of both of the secretases in the P4- or P8-treated mouse brains and *in vitro*, attesting to a significant degree of specificity of the reduction in Aβ by these peptides.

## Discussion

The evidence reported in this paper shows first that a family of peptides (Table 1) containing the DEEEDEEL sequence (P6, P7 and P8) and another independent peptide (P4), all derived from a region of the amino terminus of PS-1 that is closest to the first TMD (amino acids 41–80), are each capable of markedly reducing the production of total Aβ and of Aβ 40 and 42 *in vitro* and *in vivo* in the brains of mThy1-hAPP Tg mice. On the other hand, peptides P2 (amino acids 1–40), P5 and scrambled P1 did not reduce Aβ. Second, we show evidence using biolayer interferometry for those peptides that were effective in reducing Aβ, of a strong, specific and biologically relevant binding with the purified ectodomain of human APP 695. This binding was further confirmed by fluorescence confocal microscopy for cell-surface-expressed APP in transfected fibroblasts. Furthermore, we show evidence that strongly suggests that the binding of P4 and P8 occurs at different sites on the APP ectodomain. Finally, we demonstrate that the reduction of Aβ by the peptides does not affect the catalytic activities of β- or γ-secretase, or the level of APP. These findings have bearing on the therapeutic potential of the inhibitory peptides as discussed below.

The precise peptide binding sites on APP for P4 and P8 have not yet been mapped but we may speculate on possibilities. APP has a complex domain structure, with two highly conserved regions, E1 and E2 in its ectodomain, that is separated by an acidic region [37]. The E1 region consists of a low affinity heparan sulfate proteoglycan (HSPG) binding domain [38] and a small Cu(II) binding domain [39]. The E2 region forms an independently folded structure and contains the high-affinity HSPG binding site [40]. Crystal structure studies have shown that the negatively charged HSPG interacts with a stretch of positively charged His residues within E2 and promotes its dimerization by binding a positively charged pocket produced by two APP ectodomains arranged in *trans* [41, 42]. Whether the negatively charged P8 (but not P4) also binds APP at the same site on E2 is not known, but is likely. Unlike HSPG however, which exhibits two APP molecules for the binding, our data with biolayer interferometry show that P8 binds APP in a 1:1 ratio. P8 may function after binding to APP by preventing or promoting its homophilic interaction, in *cis* or *trans* [42,43], with another APP molecule, which in turn may impact Aβ production by directly affecting γ-secretase processing of APP [44, 45]. If P8 indeed functions by binding E2, then P4, following its interaction with APP, must exert its effect by a separate mechanism to that of P8. Crystal structure of the peptide-bound APP ectodomain will help identify the binding sites for the two peptides and shed light on further downstream events.

P4 and P8 may also function by competing for the binding of APP with PS-1. Although the catalytic site in PS-1 resides between TMDs 6 and 7 [6], it is believed to be separate from the substrate binding site and binding of PS-1 and cleavage of substrate are two separate events. Previous studies have variously identified the hydrophilic NH_2_-terminus of PS-1 [46, 47, 13], the PS-1 N-terminal fragment and PS-1 TMD 1 [48, 49] among major sites of interaction with APP (the latter two studies, however, showed an interaction of PS-1 TMD 1 with the C-terminus of APP rather than the ectodomain, as demonstrated by our current studies). Although the APP ectodomain must first be released by the action of β-or α-secretase before cleavage by γ-secretase, there is evidence from several reports of the interaction of PS with full-length APP [46, 47, 50, 51] and Notch [52]. Such interactions of different substrates with various regions of PS that lie outside the catalytic site may be a means to provide substrate selectivity for a multi-substrate enzyme with loose substrate-cleavage specificity such as γ-secretase. The endosome has been shown to be the major compartment for Aβ production in cell-lines and in the brain [53, 54] and endocytosis of APP is required for Aβ production [55]. The active γ-secretase complex has been localized in the endosome as well as the plasma membrane and it is possible that the inhibitory peptides, once bound to full-length APP may competitively inhibit its interaction with PS, as part of the γ-secretase complex, either at the cell-surface (this would require the NH_2_-terminus of PS-1 to face the exoplasm) or after internalization in the endosome. The precise manner in which peptides P4 and P8 effect substrate cleavage once bound to APP such that they prevent its further processing to Aβ, and whether the dimerization state of APP plays a role in this process, is under investigation.

Two types of technologies have largely been pursued in the field thus far in efforts to reduce the levels of Aβ: β- or γ- secretase inhibitors or modulators, and the use of Aβ-specific monoclonal antibodies. Neither approach has been entirely successful, and γ-secretase inhibitors have been particularly problematic because of off-target effects due to the inhibition of their other substrates, Notch in particular. The novel and entirely different approach to inhibit the production of Aβ described in this paper is highly advantageous, as it does not affect the catalytic activities of β- or γ-secretase and it reduces the production of Aβ specifically and substantially. Furthermore, the indication that the two best independent peptides, P4 and P8, bind to different sites on APP, suggests the possibility that they may be effective both singly or synergistically. Finally, we now have evidence that will be presented elsewhere, that P8 is stable and can be successfully delivered to the brain in rats, and is being further developed as a disease-modifying drug candidate for Alzheimer’s disease.

## References

[1] Lleo A, Greenberg SM, Growdon JH. Current Pharmacotherapy for Alzheimer’s disease. Annu Rev Med. 2006;57: 513–533. 16409164 10.1146/annurev.med.57.121304.131442

[2] Glenner GG, Wong CW. Alzheimer's disease: initial report of the purification and characterization of a novel cerebrovascular amyloid protein. Biochem Biophys Res Commun. 1984;120: 885–890. 6375662 10.1016/s0006-291x(84)80190-4

[3] Oddo S, Caccamo A, Kitazawa M, Tseng BP, LaFerla FM. Amyloid deposition precedes tangle formation in a triple transgenic model of Alzheimers disease. Neurobiol Aging. 2003;24: 1063–1070. 14643377 10.1016/j.neurobiolaging.2003.08.012

[4] Kang J, Lemaire H-G, Unterbeck J, Salbaum JM, Masters C, Grzeschik K-H et al The precursor of Alzheimer's disease amyloid A4 protein resembles a cell-surface receptor. Nature. 1987;325: 733–736. 2881207 10.1038/325733a0

[5] Takasugi N, Tomita T, Hayashi I, Tsuruoka M, Niimura M, Takahashi Y et al The role of presenilin cofactors in the gamma-secretase complex. Nature. 2003;422: 438–441. 12660785 10.1038/nature01506

[6] Ahn K, Shelton CC, Tian Y, Zhang X, Gilchrist ML, Sisodia S et al Activation and intrinsic gamma-secretase activity of presenilin 1. Proc Natl Acad Sci USA. 2010;107: 21435–21440. 10.1073/pnas.1013246107 21115843 PMC3003001

[7] Wakabayashi T, De Strooper B. Presenilins: members of the gamma-secretase quartets, but part-time soloists too. Physiology 2008;23: 194–204. 10.1152/physiol.00009.2008 18697993

[8] De Strooper B, Annaert W, Cupers P, Saftig P, Craessaerts K, Mumm JS et al A presenilin-1-dependent gamma-secretase-like protease mediates release of Notch intracellular domain. Nature. 1999;398: 518–522. 10206645 10.1038/19083

[9] He G, Luo W, Li P, Remmers C, Netzer W, Hendrick J et al Gamma-secretase activating protein is a therapeutic target for Alzheimer's disease. Nature. 2010; 467: 95–98. 10.1038/nature09325 20811458 PMC2936959

[10] Kounnas MZ, Danks AM, Cheng S, Tyree C, Ackerman E, Zhang X et al Modulation of gamma-secretase reduces beta-amyloid deposition in a transgenic mouse model of Alzheimer's disease. Neuron. 2010;67: 769–780. 10.1016/j.neuron.2010.08.018 20826309 PMC2947312

[11] Lleo A, Saura CA. γ-secretase substrates and their implications for drug development in Alzheimer’s disease. Curr Top Med Chem. 2011;11: 1513–1527. 21510835 10.2174/156802611795861004

[12] Dewji NN, Singer SJ. Genetic clues to Alzheimer’s disease. Science. 1996;271: 159–160. 8539612 10.1126/science.271.5246.159

[13] Dewji NN, Mukhopadhyay D, Singer SJ. An early specific cell-cell interaction occurs in the production of beta-amyloid in cell cultures. Proc Natl Acad Sci USA. 2006;103: 1540–1545. 16432203 10.1073/pnas.0509899103 PMC1345709

[14] Dewji NN, Valdez D, Singer SJ. The presenilins turned inside out: implications for their structures and functions. Proc Natl Acad Sci USA. 2004;101: 1057–1062. 14732691 10.1073/pnas.0307290101 PMC327150

[15] Dewji NN. The structure and functions of the presenilins. Cell Mol. Life Sci. 2005;62: 1109–1119. 15798893 10.1007/s00018-005-4566-9 PMC11138377

[16] Dewji NN, Singer SJ. Cell surface expression of the Alzheimer disease-related presenilin proteins. Proc Natl Acad Sci USA. 1997;94: 9926–9931. 9275228 10.1073/pnas.94.18.9926 PMC23298

[17] Schwarzman AL, Singh N, Tsiper M, Gregori L, Dranovsky A, Vitek MP et al Endogenous presenilin 1 redistributes to the surface of lamellipodia upon adhesion of Jurkat cells to a collagen matrix. Proc Natl Acad Sci USA. 1999;96: 7932–7937. 10393925 10.1073/pnas.96.14.7932 PMC22165

[18] Doan A, Thinakaran G, Borchelt DR, Slunt HH, Ratovitsky T, Podlisny M et al Protein topology of presenilin 1. Neuron. 1996;17: 1023–1030. 8938133 10.1016/s0896-6273(00)80232-9

[19] Li X, Greenwald I. Membrane topology of the C. elegans SEL-12 presenilin. Neuron. 1996;17: 1015–1021. 8938132 10.1016/s0896-6273(00)80231-7

[20] Lehmann S, Chiesa R, Harris DA. Evidence for a six-transmembrane domain structure of presenilin-1. J Biol Chem.1997;272: 12047–12051. 9115271 10.1074/jbc.272.18.12047

[21] Nakai T, Yamasaki A, Sakaguchi M, Kosaka K, Mihara K, Amaya Y et al Membrane Topology Of Alzheimer’s Disease-Related Presenilin 1. Evidence for the existence of a molecular species with a seven membrane-spanning and one membrane-embedded structure. J Biol Chem.1999;274: 23647–23658. 10438548 10.1074/jbc.274.33.23647

[22] Laudon H, Hansson E, Melen K, Bergman A, Farmery MR, Winblad B et al A nine-transmembrane domain topology for presenilin 1. J Biol Chem. 2005;280: 35352–35360. 16046406 10.1074/jbc.M507217200

[23] Oh YS, Turner RJ. Evidence that the COOH terminus of human presenilin 1 is located in extracytoplasmic space. Am J Physiol Cell Ph. 2005;289: 576–581. 10.1152/ajpcell.00636.2004 PMC1361293 15843437

[24] Li X, Shangyu D, Yan C, Xinqi G, Wang J, Shi Y. Structure of a presenilin family intramembrane aspartate protease. Nature. 2013;493: 56–61. 10.1038/nature11801 23254940

[25] Von Heijne G. Membrane protein topology. Nat Rev Mol Cell Bio. 2006;7: 909–918. 17139331 10.1038/nrm2063

[26] Rapp M, Granseth E, Seppala S, von Heijne G. Identification and evolution of dual topology membrane proteins. Nat Struct Mol Biol. 2006;13: 112–116. 16429150 10.1038/nsmb1057

[27] Rockenstein E, Mallory M, Mante M, Sisk A, Masliaha E. Early formation of mature amyloid-beta protein deposits in a mutant APP transgenic model depends on levels of Abeta (1–42). J Neurosci Res. 2001;66: 573–582. 11746377 10.1002/jnr.1247

[28] Franklin K, Paxinos G. The Mouse Brain in Stereotaxic Coordinates. San Diego: Academic Press; 1997.

[29] Masliah E, Rockenstein E, Veinbergs I, Sagara Y, Mallory M, Hashimoto M et al β-amyloid peptides enhance alpha-synuclein accumulation and neuronal deficits in a transgenic mouse model linking Alzheimer's disease and Parkinson's disease. Proc Natl Acad Sci USA. 2001;98: 12245–12250. 11572944 10.1073/pnas.211412398 PMC59799

[30] Mucke L, Masliah E, Yu G-Q, Mallory M, Rockenstein E, Tatsuno G et al High-level neuronal expression of Abeta 1–42 in wild-type human amyloid protein precursor transgenic mice: synaptotoxicity without plaque formation. J Neurosci. 2000;20: 4050–4058. 10818140 10.1523/JNEUROSCI.20-11-04050.2000 PMC6772621

[31] O’Reilly DR, Miller LK, Luckow V. Baculovirus Expression vectors: A laboratory Manual. New York: Freeman Press 1992 pp150–155.

[32] Verheijen JH, Hulsman LGM, van Lent N, Neumann U, Paganetti P, Hack CE et al Detection of a soluble form of BACE-1 in human cerebrospinal fluid by a sensitive activity assay. Clin Chem. 2006;52: 1168–1174. 16614000 10.1373/clinchem.2006.066720

[33] Keselman HJ, Huberty CJ, Olejnik S, Cribbie RA, Donahue B, Kowalchuk RK et al Statistical practices of educational researchers: An analysis of their ANOVA, MANOVA, and ANCOVA analyses. Rev Educ Res. 1998;68: 350–386.

[34] Rich RL, Papalia GA, Flynn PJ, Furneisen J, Quinn J, Klein JS et al A global benchmark study using affinity-based biosensors. Anal. Biochem. 2009;386: 194–216. 10.1016/j.ab.2008.11.021 19133223 PMC3793259

[35] Mumm JS, Kopan R. Notch signaling: from the outside in. Dev Biol. 2000;228: 151–165. 11112321 10.1006/dbio.2000.9960

[36] Zage PE, Nolo R, Fang W, Stewart J, Garcia-Manero G, Zweidler-McKay PA. Notch pathway activation induces neuroblastoma tumor cell growth arrest. Pediatr Blood Cancer. 2012;58: 682–689 10.1002/pbc.23202 21744479 PMC3264695

[37] Zheng H, Koo EH. Biology and Pathophysiology of the amyloid precursor protein. Mol Neurodegener. 2011; 6:27–43. 10.1186/1750-1326-6-27 21527012 PMC3098799

[38] Dahms SO, Hoefgen S, Roeser D, Schlott B, Guhrs KH, Than ME. Structure and biochemical analysis of the heparin-induced E1 dimer of the amyloid precursor protein. Proc Natl Acad Sci USA. 2010;107: 5381–5386. 10.1073/pnas.0911326107 20212142 PMC2851805

[39] Barnham KJ, McKinstry WJ, Multhaup G, Galatis D, Morton CJ, Curtain CC et al Structure of the Alzheimer’s disease amyloid precursor protein copper-binding domain—a regulator of neuronal copper homeostasis. J Biol Chem. 2003;278: 17401–17407. 12611883 10.1074/jbc.M300629200

[40] Gralle M, Oliveira LP, Guerreiro LH, McKinstry WJ, Galatis D Masters CL. et al Solution conformation and heparin-induced dimerization of the full-length extracellular domain of the human amyloid precursor protein. J Mol Bio. 2006;357: 493–508. 16436282 10.1016/j.jmb.2005.12.053

[41] Lee S, Xue Y, Hu J, Wang Y, Lui X, Demeler B et al The E2 domains of APP and APLP1 share a conserved mode of dimerization. Biochemistry. 2011;50: 5453–5464. 10.1021/bi101846x 21574595 PMC3120129

[42] Wang Y, Ha Y. The X-ray structure of an antiparallel dimer of the human amyloid precursor protein E2 domain. Mol Cell. 2004;15: 343–353. 15304215 10.1016/j.molcel.2004.06.037

[43] Scheuermann S, Hambsch B, Hesse L, Stumm J, Schmidt C, Beher D, et al Homodimerization of amyloid precursor protein and its implication in the amyloidogenic pathway of Alzheimers disease. J Biol Chem. 2001;276: 33923–33929. 11438549 10.1074/jbc.M105410200

[44] So P, Zeldich E, Seyb KI, Huang MM, Concannon JB, King GD et al Lowering of amyloid β peptide production with a small molecule inhibitor of amyloid-β precursor protein dimerization. Am J Neurodegener Disease. 2012;1: 75–87. 22822474 PMC3560454

[45] Eggert S, Midthune B, Cottrell B, Koo EH. Induced dimerization of the amyloid precursor protein leads to decreased amyloid-beta protein production. J Biol Chem. 2009;284: 28943–28952. 10.1074/jbc.M109.038646 19596858 PMC2781440

[46] Dewji NN, Singer SJ. Specific transcellular binding of membrane proteins crucial to Alzheimer’s disease. Proc Natl Acad Sci USA. 1996;93: 12575–12580. 8901624 10.1073/pnas.93.22.12575 PMC38034

[47] Pradier L, Carpentier N, Delalonde L, Clavel N, Bock MD, Buee L et al Mapping the APP/presenilin (PS) binding domains: the hydrophilic N-terminus of PS-2 is sufficient for interaction with APP and can displace APP/PS-1 interaction. Neurobiol Dis. 1999;6: 43–55. 10078972 10.1006/nbdi.1998.0212

[48] Annaert WG, Esselens C, Baert V, Doeve C, Snellings G, Cupers P et al Interaction with telencephalin and the amyloid precursor protein predicts a ring structure for presenilins. Neuron. 2001;32: 579–589. 11719200 10.1016/s0896-6273(01)00512-8

[49] Takagi S, Tominaga A, Sato C, Tomita T, Iwatsubo T. Participation of the transmembrane domain 1 of Presenilin1 in the catalytic pore structure of the γ-secetase. J Neurosci. 2010;301: 15943–15950. 10.1523/JNEUROSCI.3318-10.2010 PMC6633739 21106832

[50] Waragai M, Imafuku I, Takeuchi S, Kanazawa I, Oyama F, Udagawa Y et al Presenilin-1 binds to amyloid precursor protein directly. Biochem Biophys Res Commun. 1997;239: 480–482. 9344855 10.1006/bbrc.1997.7488

[51] Weidemann A, Pliga K, Durrwang U, Czech C, Evin G, Masters C et al Formation of stable complexes between two Alzheimer’s disease gene products: presenilin-2 and β-amyloid precursor protein. Nat Med. 1997;3: 328–332. 9055862 10.1038/nm0397-328

[52] Ray WJ, Yao M, Nowotny P, Mumm J, Zhang W, Wu J et al Evidence for a physical interaction between presenilin and Notch. Proc Natl Acad Sci USA. 1999;96: 3263–3268. 10077672 10.1073/pnas.96.6.3263 PMC15930

[53] Cataldo AM, Barnett JL, Pieroni C and Nixon RA. Increased neuronal endocytosis and protease delivery to early endosomes in sporadic Alzheimers disease: neuropathologic evidence for a mechanism of increased β-amyloidogenesis. J Neurosci. 1997;17: 186–199. 10.1523/JNEUROSCI.17-16-06142.1997 PMC6568334 9236226

[54] Nixon RA. Endosome function and dysfunction in Alzheimer’s disease and other neurodegenerative diseases. Neurobiol Aging. 2005;26: 373–382. 15639316 10.1016/j.neurobiolaging.2004.09.018

[55] Cirrito JR, Kang J-E, Lee J, Stewart FR, Verges DK, Silverio LM et al Endocytosis is required for synaptic-activity-dependent release of amyloid-β in vivo. Neuron. 2008;58: 42–51. 10.1016/j.neuron.2008.02.003 18400162 PMC2390913

