# Expression of Concern: Peptides of presenilin-1 bind the amyloid precursor protein ectodomain and offer a novel and specific therapeutic approach to reduce β-amyloid in Alzheimer’s disease

**DOI:** 10.1371/journal.pone.0319769

**Published:** 2025-02-27

**Authors:** The PLOS One Editors

Following the publication of this article [1], concerns were raised regarding results presented in Fig 7. Specifically,

**Fig 7:**
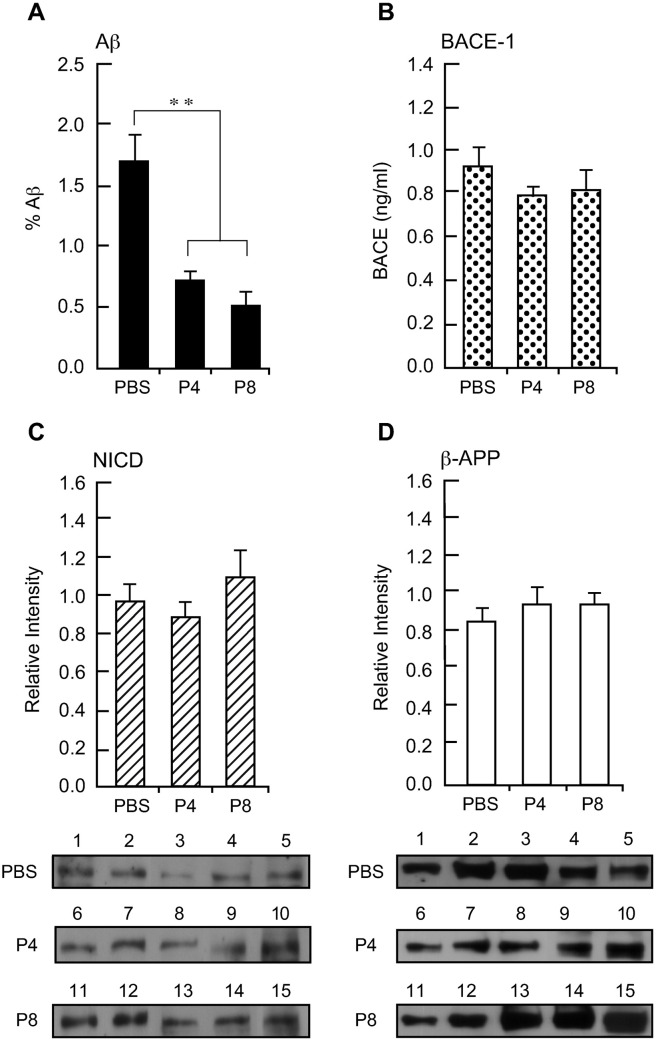
Effect of peptides P4 and P8 treatment on levels of Aβ, APP, NICD and BACE-1 in neocortex of APP Tg mice. A. Aβ levels in sections of mouse neocortex that was immunolabeled with a MAb against human Aβ1–16 were quantified as in Fig 2. Data are expressed as mean ± s.e.m. n =  5. ** *p* < 0.005. **B.** BACE-1 activity in extracts of neocortex of mice treated with peptides P4, P8 and PBS was determined using the SensiZyme BACE1 activity assay kit. Absorbance was monitored at 405 nm. Data are expressed as mean ± s.e.m. of active BACE-1 in ng/ml n =  5. **C.** Extracts of neocortex of peptide-treated mice were Western-blotted with primary rabbit Ab against NICD followed by HRP-conjugated goat anti-rabbit IgG. Immunoreactive bands were detected by ECL and the signal intensity of the protein bands was quantified. Top: data are expressed as mean ± s.e.m. n =  5. Bottom: The individual Western blot gel images showing immunoreactive bands. **D.** After NICD detection, the nitrocellulose membranes were stripped and re-probed with a MAb against APP, followed by HRP-conjugated goat anti-mouse IgG. The signal intensity of the protein bands was then quantified. Top: data are expressed as mean ± s.e.m. n =  5. Bottom: The individual Western blot gel images showing immunoreactive bands.

- Fig 7C PBS panel, there appears to be a splice line between lanes 2 and 3.
- The Fig 7C P4 panel appears similar to the Fig 7D P4 panel, despite being used to represent different experimental conditions.

The corresponding author confirmed that the Fig 7C P4 panel was inadvertently duplicated during figure preparation and erroneously presented in Fig 7D. They provided a replacement Fig 7D P4 panel as well as individual-level data underlying the graphs presented in Figs 7C and 7D (S1 File).

The authors provided cropped image data to support the results presented in Fig 7C and 7D (S1 File), which confirm that most if not all blot panels presented in Fig 7 report spliced image data, i.e., each blot panel in Fig 7 includes data from at least two different blot images in S1 File. There are also several instances in which Fig 7C and 7D lane labels imply results are from the same biological replicate whereas the labels in S1 File indicate otherwise. These issues call into question the reliability of results drawn from comparing results within and across blots in Fig 7C and Fig 7D.

Furthermore, the corresponding author stated that equal amounts of protein per sample was loaded onto the gels for the Fig 7C and Fig 7D experiments, but the experimental design did not include control blots to confirm equal loading or transfer. Instead, the extract of mouse 2026 was loaded onto all gels and quantification data across different blots were normalized to the 2026 results. The journal is concerned about the reliability of the western blot results given this study design issue (no loading control blots).

Contrary to the article’s Data Availability statement, the data underlying the published results were not provided with the article. The available data are provided in the S1–S6 Files included with this notice.

The *PLOS One* Editors issue this Expression of Concern to notify readers of the above issues and relay the available data provided by the corresponding author. Results reported in Fig 7C and 7D should be interpreted with caution.

## Supporting information

S1 File Underlying data provided for Fig 7C and 7D. (XLS)

S2 File Available data provided for Fig 1. (ZIP)

S3 File Available data provided for Fig 2. Note, the “Date” data in column E appear to be erroneous. (ZIP)

S4 File Available data provided for Fig 4. (ZIP)

S5 File Available data provided for Fig 7A and 7B. (ZIP)

S6 File Available data provided for Fig 8. (ZIP)
