# Supplementary material for: Expression of Concern: Peptides of presenilin-1 bind the amyloid precursor protein ectodomain and offer a novel and specific therapeutic approach to reduce β-amyloid in Alzheimer’s disease
Source: PLoS One. 2025 Feb 27;20(2):e0319769. doi: 10.1371/journal.pone.0319769 (PMC11867307; doi:10.1371/journal.pone.0319769)
Supplement: S2 File — (ZIP) [file pone.0319769.s002.zip › Fig 1AC.pdf]

11/6/14

Final Aß40 Values

P1		1		2	Average % Aß	SD	Variance
0	100		100		100	0	0
2	95		115		105	14	200
4	100		105		103	4	13
5	100		105		103	4	13

SP1							
0	100	100	73	80	88	14	192
2	100	115	100	100	104	8	56
4	139	118	90	96	111	22	500
5	106	103			105	2	5

P2				
0	100	100	100	0	0
2	136	60	98	54	2888
4	124	90	107	24	578
5	76	130	103	38	1458

P3					
0	100	100	100	0	0
2	80	100	90	14	200
4	58	52	55	4	18
5	41	68	55	19	365

P4					
0	100	100	100	0	0
2	75	100	88	18	313
4	80	36	58	31	968
5	70	34	52	25	648

P5

0	100	100	100	0	0
2	92	92	92	0	0
4	88	96	92	6	32
5	112	116	114	3	8

P6

0	100	100	100	0	0
2	64	73	69	6	41
4	59	73	66	10	98
5	59	53	56	4	18

P7

0	100	100	100	0	0
2	70	86	78	11	128
4	64	60	62	3	8
5	58	40	49	13	162

P8

0	100	76	100	92	14	192
2	73	69	86	76	9	79
4	52	73		63	15	221
5	47	54	43	48	6	31

P9

0	100	100	100	0	0
2	95	115	105	14	200
4	95	100	98	4	13
5	109	100	104	6	41

P10

0	100	100	100	0	0
2	95	95	95	0	0
4	95	95	95	0	0
5	104	100	102	3	8
