# Supplementary material for: Expression of Concern: Peptides of presenilin-1 bind the amyloid precursor protein ectodomain and offer a novel and specific therapeutic approach to reduce β-amyloid in Alzheimer’s disease
Source: PLoS One. 2025 Feb 27;20(2):e0319769. doi: 10.1371/journal.pone.0319769 (PMC11867307; doi:10.1371/journal.pone.0319769)
Supplement: S2 File — (ZIP) [file pone.0319769.s002.zip › Fig 1BD.pdf]

11/6/14

Final Aß 42 Values

P1	1	2	Average	SD	Variance
			% Aß		
0	100	100	100	0	0
2	85.5	64	75	15	231
4	85.5	72	79	10	91
5	83.8	80	82	3	7

SP1

0	100	100	100	0	0
2	100	X	100	0	0
4	100	85	93	11	113
5	120	92	106	20	392

P2

0	100	100	100	0	0
2	127	111	119	11	128
4	111	116	114	4	13
5	111	111	111	0	0

P3

0	100	96	98	3	8
2	37	60	49	16	265
4	55	16	36	28	761
5	19	20	20	1	1

P4

0	100	X	100	X	X
2	78	76	77	1	2
4	63	76	69	10	91
5	85	53	69	23	512

P5

0	100	X	100	X	X
2	100	100	100	0	0
4	100	100	100	0	0
5	121	81	101	28	800

P6

0	100	100	100	0	0
2	52	80	66	20	392
4	48	70	59	16	242
5	45	70	58	18	313

P7

0	100	100	100		100	0	0
2	62.5	71	75	100	77	16	260
4	35	60			48	18	313
5	55	26			41	21	421

P8

0	100	100	100	0	0
2	44	48	46	3	8
4	43	44	44	0	0
5	36	61	49	18	313

P9

0	100	100	100	100	100	0	0
2	133	92	100	105	108	18	318
4	133	130	100	100	116	18	332
5	120	125	105	105	114	10	106

P10

0	100	100	100	100	100	0	0
2	67	116	105	112	100	22	505
4	67	141	100	100	102	30	918
5	56	108	105	102	93	25	606
