# Supplementary material for: Expression of Concern: Peptides of presenilin-1 bind the amyloid precursor protein ectodomain and offer a novel and specific therapeutic approach to reduce β-amyloid in Alzheimer’s disease
Source: PLoS One. 2025 Feb 27;20(2):e0319769. doi: 10.1371/journal.pone.0319769 (PMC11867307; doi:10.1371/journal.pone.0319769)
Supplement: S3 File — (ZIP) [file pone.0319769.s003.zip › Cortex and Hippocampus.pdf]

% Aβ	Cortex							
	PBS	P1	P2	P3	P4	P8	P9	P10
	1.21	0.7	1.1	0.7	0.615	0.31	1.62	0.31
	2.51	0.9	0.9	0	1.123	1.33	0.7726	1.421
	1.61	1.3	1.2	0.1	0.762	0.242	0.445	0.912
	1.671			1.679	1.32	0.812	1.324	0.928
	0.923			1.14	0.5126	0.76	0.7236	0.71
	1.67			1.245	0.716	0.47	0.65	1.142
	2.28				1.13	0.67		
	1.541				0.965	0.851		
	1.431				0.751	0.937		
	1.8							
	0.8							
	0.5							

% Aβ	Hippocampus							
	PBS	P1	P2	P3	P4	P8	P9	P10
	0.9182	0.3	0.6	0.3	0.514	0.213	1.312	0.52
	2.134	0.7	0.2	0	1.41	1.223	0.83	1.162
	1.425	0.9	0.7	0.1	0.8526	0.341	0.3162	0.82
	1.552			1.12	1.134	0.772	1.726	0.781
	1.12			0.975	0.425	0.79	0.423	0.6152
	1.39			0.765	0.625	0.531	0.72	1.524
	1.651				0.76	0.33		
	1.1				0.342	0.291		
	0.932				0.452	0.225		
	0.8							
	0.2							
	0.2							
