# Supplementary material for: Expression of Concern: Peptides of presenilin-1 bind the amyloid precursor protein ectodomain and offer a novel and specific therapeutic approach to reduce β-amyloid in Alzheimer’s disease
Source: PLoS One. 2025 Feb 27;20(2):e0319769. doi: 10.1371/journal.pone.0319769 (PMC11867307; doi:10.1371/journal.pone.0319769)
Supplement: S3 File — (ZIP) [file pone.0319769.s003.zip › Mouse data Figure 2.pdf]

Line	Mouse#	Room#	DOB	DOS	Age	Sex	Wks	DNA	DNA	Color	Tx	RR	OF	Brain	Frz	Remarks
41B	1922	154	1/30/09	10/12/09	8.38	m	36.3	+		bl	10			1/2Frz,1/2Fix		
41B	1923	154	1/30/09	10/12/09	8.38	m	36.3	+		bl	10			1/2Frz,1/2Fix		
41B	1941	154	2/9/09	10/12/09	8.05	m	34.9	+		bl	PBS			1/2Frz,1/2Fix		
41B	1946	154	2/17/09	10/12/09	7.79	m	33.8	+		bl	8			1/2Frz,1/2Fix		
41B	1966	154	3/11/09	10/12/09	7.07	m	30.6	+		bl	9			1/2Frz,1/2Fix		
41B	1974	154	3/13/09	10/12/09	7.00	m	30.3	-		bl	PBS			1/2Frz,1/2Fix		
41B	1988	154	4/1/09	10/12/09	6.38	m	27.6	+		bl	8			1/2Frz,1/2Fix		
41B	1991	154	4/4/09	10/12/09	6.28	f	27.2	+		3	PBS			1/2Frz,1/2Fix		
41B	1992	154	4/4/09	10/12/09	6.28	f	27.2	+		3	4			1/2Frz,1/2Fix		
41B	1993	154	4/4/09	10/12/09	6.28	f	27.2	-		bl	PBS			1/2Frz,1/2Fix		
41B	1994	154	4/4/09	10/12/09	6.28	f	27.2	+		3	8			1/2Frz,1/2Fix		
41B	1995	154	4/4/09	10/12/09	6.28	f	27.2	+		3	9			1/2Frz,1/2Fix		
41B	2000	154	4/4/09	10/12/09	6.28	m	27.2	+		bl	9			1/2Frz,1/2Fix		
41B	2003	154	4/4/09	10/12/09	6.28	m	27.2	+		bl	10			1/2Frz,1/2Fix		Cannula found pulled out 9/21
41B	2005	154	4/4/09	10/12/09	6.28	m	27.2	+		bl	4			1/2Frz,1/2Fix		
41B	2007	154	4/4/09	10/12/09	6.28	m	27.2	+		3	10			1/2Frz,1/2Fix		
41B	2008	154	4/4/09	10/12/09	6.28	m	27.2	+		3	4			1/2Frz,1/2Fix		
41B	2009	154	4/4/09	10/12/09	6.28	m	27.2	+		3	4			1/2Frz,1/2Fix		
41B	2018	154	4/4/09	10/12/09	6.28	m	27.2	+		3	4			1/2Frz,1/2Fix		
41B	2019	154	4/4/09	10/12/09	6.28	m	27.2	+		3	PBS			1/2Frz,1/2Fix		
41B	2020	154	4/7/09	10/12/09	6.18	f	26.8	+		3	9			1/2Frz,1/2Fix		
41B	2024	154	4/7/09	10/12/09	6.18	f	26.8	+		3	8			1/2Frz,1/2Fix		
41B	2026	154	4/7/09	10/12/09	6.18	m	26.8	+		bl	8			1/2Frz,1/2Fix		
41B6	457	154	1/29/09	10/12/09	8.42	m	36.5	+		bl	9			1/2Frz,1/2Fix		
41B6	462	154	2/7/09	10/12/09	8.12	m	35.2	+		bl	PBS			1/2Frz,1/2Fix		
41B6	463	154	2/7/09	10/12/09	8.12	m	35.2	+		bl	PBS			1/2Frz,1/2Fix		
41B6	464	154	2/7/09	10/12/09	8.12	m	35.2	-		bl	PBS			1/2Frz,1/2Fix		
41B6	470	154	2/10/09	10/12/09	8.02	m	34.8	+		bl	8			1/2Frz,1/2Fix		
41B6	476	154	2/27/09	10/12/09	7.46	m	32.3	+		bl	4			1/2Frz,1/2Fix		
41B6	486	154	4/3/09	10/12/09	6.31	m	27.4	+		bl	9			1/2Frz,1/2Fix		
41B6	487	154	4/4/09	10/12/09	6.28	f	27.2	+		3	PBS			1/2Frz,1/2Fix		Cannula found pulled out after 14 days
41B6	503	154	4/7/09	10/12/09	6.18	m	26.8	+		bl	10			1/2Frz,1/2Fix		
41B	1973	154	3/13/09	9/14/09	6.08	m	26.4	+		bl	10			FD 9/14/09		
Dewji Experiment																
Start 9/10/09																
	Injected peptide by way of cannula and osmotic pump into ventricle (-1.0,1.0,-3.0) (from bregma, lateral, into)															
	5 compounds used; peptide 4, peptide 8, peptide 9, peptide 10, and PBS.															
Sacrificed 10/12/09																
