# Supplementary material for: Expression of Concern: Peptides of presenilin-1 bind the amyloid precursor protein ectodomain and offer a novel and specific therapeutic approach to reduce β-amyloid in Alzheimer’s disease
Source: PLoS One. 2025 Feb 27;20(2):e0319769. doi: 10.1371/journal.pone.0319769 (PMC11867307; doi:10.1371/journal.pone.0319769)
Supplement: S5 File — (ZIP) [file pone.0319769.s005.zip › Normalised data Fig 7A.pdf]

Individual level data for Fig. 7A (Aß)

PBS

Mouse %Aß Cortex Mean: 1.7

2019	1.7
1941	1.7
462	1.2
487	1.6
1605	2.3

P4

Mouse %Aß Cortex Mean: 0.7

476	0.6
2009	0.5
2018	0.7
1634	0.97
2005	0.8

P8

Mouse %Aß Cortex Mean: 0.5

470	0.3
1988	0.2
1606	0.7
1628	0.9
2026	0.5
