# Supplementary material for: Expression of Concern: Peptides of presenilin-1 bind the amyloid precursor protein ectodomain and offer a novel and specific therapeutic approach to reduce β-amyloid in Alzheimer’s disease
Source: PLoS One. 2025 Feb 27;20(2):e0319769. doi: 10.1371/journal.pone.0319769 (PMC11867307; doi:10.1371/journal.pone.0319769)
Supplement: S6 File — (ZIP) [file pone.0319769.s006.zip › Underlying data Fig 8.pdf]

NICD

Inhibitor (nM)			Average	Std. Dev.
0	100	100	100	0
1	94	84	89	6.89
3	87	82	85	3.44
10	82	71	77	7.77
30	71	61	66	7.09
100	X	56	56	#DIV/0!
**P8 (µM)**			**Average**	**Std. Dev.**
0	100	100	100	0
0.5	95	100	97	3.39
1	94	101	97	5.32
2	88	93	91	3.63
5	94	101	98	4.96
10	101	111	106	6.63
**P4 (µM)**			**Average**	**Std. Dev.**
0	100	100	100	0
0.5	107	107	107	0.09
1	107	104	106	2.12
2	102	100	101	1.41
5	101	99	100	1.41
10	105	102	104	2.12
**SP 1 (µM)**			**Average**	**Std. Dev.**
0	100	100	100	0.00
0.5	103	99	101	2.83
1	101	99	100	1.41
2	104	105	105	0.71
5	100	100	100	0.00
10	104	97	101	5

Data for Fig 8b

Data for Fig 8a

BACE FINAL

SP 1 (μM)			Average	Std. Dev.
**0**	100	100	100	0
**1**	100	104	102	2.8
**2**	89	118	103.5	20.5
**3**	100	113	106.5	9.2
**4**	100	114	107	9.9
**5**	89	104	96.5	10.6
**P4 (μM)**			**Average**	**Std. Dev.**
**0**	100	100	100	0
**1**	100	100	100	0
**2**	111	100	105	7.8
**3**	100	100	100	0
**4**	88	100	94	8.5
**5**	111	90	101	14.8
**BACE Inhibitor (nM)**			**Average**	**Std. Dev.**
**0**				
**100**	100	100	100	0
**200**	36	42	39	4.2
**300**	36	47	41	7.8
**400**	36	19	27.5	12.0
**500**	16	23	17.5	4.9
**P8 (μM)**			**Average**	**Std. Dev.**
**0**	100	100	100	0
**1**	107	108	107	0.70
**2**	110	103	106	4.9497475
**3**	108	105	106	2.12
**4**	111	99	105	8.49
**5**	106	102	104	2.83

Data for Fig 8c

Data for Fig 8d

Fig 8c
